# Evaluation of time to sputum smear conversion and its association with treatment outcomes among drug-resistant tuberculosis patients: a retrospective record-reviewing study

**DOI:** 10.3389/fphar.2024.1370344

**Published:** 2024-06-05

**Authors:** Abdulaziz Ibrahim Alzarea, Amna Saifullah, Yusra Habib Khan, Adullah Salah Alanazi, Ahmed D. Alatawi, Majed Ahmed Algarni, Ziyad Saeed Almalki, Abdullah K. Alahmari, Hassan H. Alhassan, Tauqeer Hussain Mallhi

**Affiliations:** ^1^ Department of Clinical Pharmacy, College of Pharmacy, Jouf University, Sakaka, Saudi Arabia; ^2^ Institute of Pharmacy, Faculty of Pharmaceutical and Allied Health Sciences, Lahore College for Women University, Lahore, Pakistan; ^3^ Department of Clinical Pharmacy, College of Pharmacy, Taif University, Taif, Saudi Arabia; ^4^ Department of Clinical Pharmacy, College of Pharmacy, Prince Sattam Bin Abdulaziz University, Al-Kharj, Saudi Arabia; ^5^Department of Clinical Laboratory Sciences, College of Applied Medical Sciences, Jouf University, Sakaka, Saudi Arabia

**Keywords:** tuberculosis, resistant-tuberculosis, sputum smear, culture, treatment outcomes, pharmacotherapy

## Abstract

**Background:** This study examined the time to sputum smear and culture conversion and determinants of conversion, as well as variables associated with treatment outcomes among drug-resistant pulmonary tuberculosis (DR-PTB) cases.

**Methods:** The electronic database and written medical records of patients were utilized to assess the sociodemographic, clinical, microbiological, and treatment characteristics and outcomes of study participants.

**Results:** Among 736 patients with pulmonary tuberculosis (PTB), the mean age was 36.5 ± 16.5 years, with males comprising 53.4% and a mean weight of 47.76 ± 11.97 kg. The median time period for sputum smear conversion and sputum culture conversion was a month. The first-month culture conversion (*p* < 0.001, aOR = 5.817, and 95% CI = 3.703–9.138) was the determinant of sputum smear conversion and receiver operating curve analysis with AUC = 0.881, 95% CI = 0.855–0.907, and *p* < 0.001, which showed a high level of predictive ability for the regression model for the initial sputum smear conversion. However, the first-month sputum conversion (*p* < 0.001, aOR = 7.446, and 95% CI = 4.869–11.388) was attributed to sputum culture conversion, and the model has shown excellent predictive ability for regression with ROC curve analysis demonstrating AUC = 0.862, 95% CI = 0.835–0.889, and *p* < 0.001. A total of 63.2% of patients showed favorable treatment outcomes, with 63.1% of cases achieving treatment-cured status. The previous use of SLD, history of smoking, duration of illness ≤ 1 year, extensively drug-resistant tuberculosis, and first-month sputum conversion were the variables attributed to favorable treatment outcomes observed in drug-resistant pulmonary tuberculosis cases. ROC curve analysis with AUC = 0.902, 95% CI = 0.877–0.927, and *p* < 0.001) has shown outstanding ability for regression model prediction for the variables influencing treatment outcomes.

**Conclusions:** Within 2 months of treatment, most patients had converted their sputum cultures and sputum smears. The determinants of early sputum smear and sputum culture conversion, as well as favorable treatment outcomes, were identified. These factors should be considered during the design and implementation of effective strategies for drug-resistant tuberculosis control programs.

## Introduction

According to the World Health Organization (WHO) tuberculosis (TB) report, the incidence rate of TB has increased by 3.6% in 2021 compared to the previous year. It has indicated a reversal of the past trend of roughly a 2% drop every year for 2 decades (Organization, 2021). The prevalence of drug-resistant tuberculosis (DR-TB) has increased in the last 15 years, especially in low-income countries, despite the fact that the inexpensive directly observed therapy (DOT) strategy greatly aids many global nations in combating and preventing the disease. This increase is primarily caused by a lack of efficient screening and treatment accessibility (Zhdanova et al., 2021; Gamachu et al., 2022). DR-TB disease is a global concern for all TB programs, with antimicrobial resistance accounting for almost one-third of all TB deaths. According to the global TB report, multi-drug resistance/rifampicin-resistant TB (MDR/RR-TB) is identified in 3%–4% of newly diagnosed cases and 18%–21% of patients with previous treatment, with the most occurrences in Asia and Africa (Chakaya et al., 2022; Gamachu et al., 2022). In Pakistan, the prevalence of pulmonary TB (PTB) is high, with the sixth highest number of TB cases in the world (Aftab et al., 2021; Khan et al., 2022). The country has 210 million inhabitants, 1.5 million of whom have TB, including 15,000 cases of MDR-TB (Ali et al., 2020; Khan et al., 2022). A comparatively high rate of RR-TB (4.2%) was discovered in new TB patients upon contrasting the old and new sources of evidence, and this percentage increased by nearly four times in cases that had previously received treatment (SHEIKH et al., 2020; Khan et al., 2022). Furthermore, DR-TB has a major economic impact as well as psychological and social stigma. As a result, DR-TB problems not only affect individual patients and their relatives but also healthcare organizations that are meant to avoid and manage their consequences. It is uncertain how long a drug-resistant PTB patient will continue to spread infection following the implementation of an effective treatment.

The conversion of positive sputum smear and culture results to negative is one of the most crucial measures of an anti-TB therapy plan’s success. Clinicians frequently utilize this to decide the duration of second- or third-line injectable agents and the overall length of the treatment (Shibabaw et al., 2018). The WHO and National TB Control Program (NTP) recommend monthly sputum smear and culture examinations for patients receiving therapy with proven pulmonary DR-TB regimens to predict treatment efficacy and track the occurrence of therapeutic failure.

The prediction of time to smear and culture conversion is crucial to establishing the guidelines for respiratory isolation. A shift in the positive sputum smear test results to negative or not detected at the start of therapy indicates a favorable TB treatment response. The first or one-month result is highly helpful in selecting whether to switch to other alternative medications that might culminate in a better endpoint treatment outcome. Clinicians and researchers should quickly identify cases that are far more prone to show unfavorable outcomes and then enhance treatment or intervention measures to help them improve overall treatment results (Gamachu et al., 2022).

To the best of our knowledge, no previous research has ever been conducted on the time to sputum smear and culture conversion, determinants of first-month sputum smear and culture conversion, and variables associated with treatment outcomes in patients with DR-TB in Pakistan. The main objective of this study is to assess the rates of sputum smear and culture conversion and determine the predictors of first-month sputum smear and culture conversion and variables related to treatment outcomes in DR-PTB patients.

## Methodology

### Ethics statement

The Allama Iqbal Memorial Hospital’s (AIMTH) Office of the Medical Superintendent granted the study approval (registration number: AIMTH/IEB/2018/564, 8566/AIMTH/SKT). The office waived the requirement for patient consent because of the retrospective study. Directly observed treatment short course (DOTS) facilitators, programmatic management of drug-resistant tuberculosis (PMDT) data managers, and attending physicians regularly gathered, recorded, and managed all the pertinent data of research participants.

### Study setting

Sialkot is one of the most important districts in the Punjab region of Pakistan. It is in the northeastern part of the province and has a land area of 3,016 square kilometers and 3.894 million inhabitants. The current investigation was successfully carried out at the AIMTH in the Pulmonology Department. The AIMTH is a teaching hospital in Sialkot district with 400 beds. Patients are either self-driven or referred by physicians from other healthcare facilities and local clinics in the city or surrounding territories.

### Study population

All patients with a confirmed diagnosis of DR-TB of pulmonary origin during the study period from October 2014 to August 2023 were included in this study. The timeframe of patient recruitment was chosen due to the hospital’s establishment of PMDT in 2014, which is an advancement in supporting the new STOP TB approach.

### Inclusion/exclusion criteria

**FIGURE 1:**
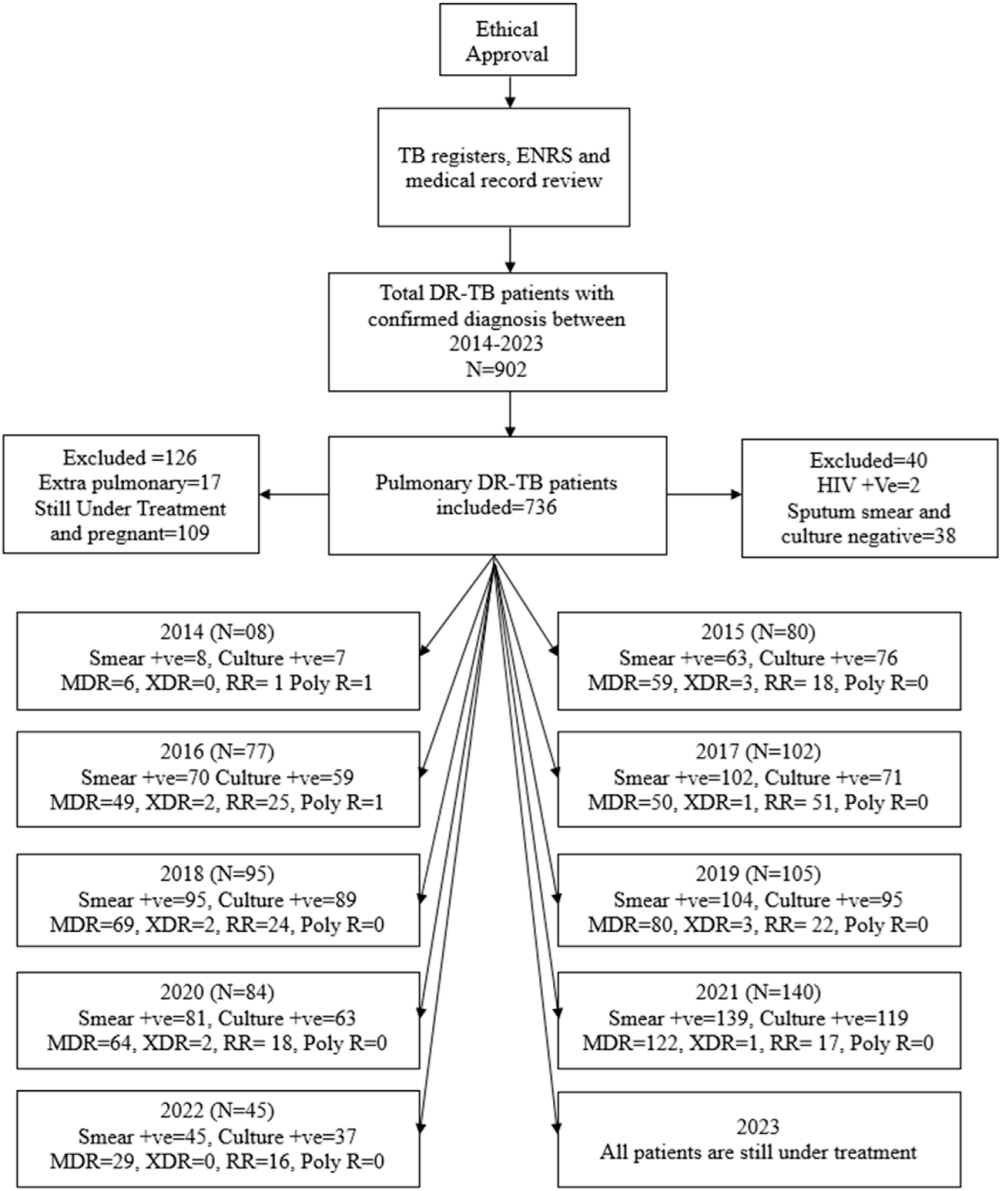
Methodological flow chart of the study. Abbreviations: TB, tuberculosis; ENRS: electronic nominal recording reporting system; DR-TB, drug-resistant tuberculosis; HIV: human immunodeficiency virus; +ve, positive; MDR, multidrug resistance; XDR, extensively drug resistance; RR, rifampicin resistance; poly R, poly-drug resistance.

In the present study, all patients aged 11 years or above, belonging to either sex, and confirmed by Xpert MTB/RIF and drug susceptibility testing (DST) as having DR-TB were included, as shown in Figure 1. However, the patients with either sputum culture- or sputum smear-positive results were also included in the final analysis.

The exclusion criteria included patients with both negative sputum smear and sputum culture results, incomplete medical/microbiological records, those currently undergoing treatment, pregnant females, and patients with HIV. The HIV patients were omitted from the study because many of them were referred to other specialty care institutions. Furthermore, pregnant women were intentionally excluded to eliminate bias caused by physiological adaptations during pregnancy.

### Data collection and management

The electronic nominal recording reporting system (ENRS) and the medical records of patients were used to extract relevant information. The sociodemographic characteristics, including age, weight, gender, residential situation, occupation, social history, and clinical characteristics of patients, including the previous course of treatment and outcomes, were collected from the electronic and medical records. The microbiological findings and gradings of the baseline sputum smear and culture and follow-up results were also noted.

### Bacteriology and DST

All the presumptive TB cases with confirmed chest X-rays were subjected to smear microscopy and rapid DST. The monthly collected sputum samples were strained and examined under a 100-times magnifying objective lens using the Ziehl–Neelsen method to identify the existence of acid-fast bacilli (AFB). The sputum smear AFB load was graded based on the presence of bacilli observed in every hundred fields. The negative values showed no AFB, while the positive values indicated scanty (1–9), 1+ (10–99), 2+ (100–990), and 3+ (>1,000) bacilli per 100 HPF (Manalebh et al., 2015; Gamachu et al., 2022; Bogale et al., 2021).

Moreover, the monthly sputum cultures were also isolated in the Lowenstein–Jensen (LJ) solid medium and were graded as negative (no growth), scanty (1-9 colonies), 1+ (10–100 colonies), 2+ (<100–200 colonies), and 3+ (>200 colonies) (Weldemhret et al., 2023).

The resistance to isoniazid and rifampicin was detected using the line probe assay (LPA), while the genotypic resistance to rifampicin was also identified and detected by Xpert MTB/RIF. The samples with positive grading were further sent for DST against all the first- and second-line anti-TB drugs (FLD and SLD). The anti-TB drugs in concentrations: isoniazid (0.2 μg/mL), rifampicin (1 μg/mL), ethambutol (5 μg/mL), streptomycin (2 μg/mL), amikacin (4 μg/mL), kanamycin (5 μg/mL), capreomycin (4 μg/mL), ofloxacin (2 μg/mL), levofloxacin (1 μg/mL), and ethionamide (5 μg/mL) were used to conduct DST using the agar proportion method on enriched Middlebrook 7H10 medium (BBL, BD, Sparks, MD, USA) (Abubakar et al., 2021). A BACTEC Mycobacterial Growth Indicator Tube (MGIT, BD, Sparks, MD, United States) was used to conduct the DST of pyrazinamide at a concentration of 100 μg/mL (Organization, 2014; Javaid et al., 2018). Based on the findings of DST, the patients were classified into different resistance groups and treated accordingly.

### Case definitions

MDR-TB: *Mycobacterium tuberculosis (MTB)* strains resistant to rifampicin and isoniazid (Wang et al., 2014; Saifullah et al., 2021). Pre-extensively drug-resistant TB (XDR-TB): RR/MDR TB patients with added resistance to any of the fluoroquinolones and are classified as Pre-XDR-TB cases. RR-TB: MTB strains resistant to rifampicin only (Saifullah et al., 2021). XDR-TB: RR/MDR TB patients with added resistance to fluoroquinolones and at least one additional group A drug such as linezolid and bedaquiline (Saifullah et al., 2021; Horne et al., 2010). Poly-drug resistant: resistance to more than a single FLD in addition to rifampicin and isoniazid.

Treatment groups: in compliance with the guidelines of the WHO, treatment registration groups and outcomes were defined as follows: New: a suspected or confirmed TB patient without any prior history of treatment or treatment for not more than a month (Saifullah et al., 2021). Previously treated: the patient who had previously taken the anti-TB treatment for a month or more (Saifullah et al., 2021). The new or previously treated patients can have either positive or negative bacteriology. Relapse: a previously treated patient declared as completed or cured but presented with a positive smear again (Saifullah et al., 2021). Previously treated after failure: category I (CAT I) cases that exhibit a positive sputum smear at the end of the fifth month, two or more consecutive positive cultures during the intensive phase, or any one of the positive cultures among the last three cultures at the completion of DR-TB treatment (Saifullah et al., 2021). Previously treated after lost to follow-up (LFU) (defaulter): previously treated cases with an interrupted therapy of 2 or more consecutive months upon the completion of their most recent regimen of treatment (Saifullah et al., 2021). Others previously treated: cases with unknown or undocumented treatment outcomes for their most current treatment course (Saifullah et al., 2021).

### Treatment outcomes

Treatment outcomes are defined and categorized according to the WHO guidelines (Alemu et al., 2020; Gamachu et al., 2022; Chakaya et al., 2022). Cured: treatment completion with at least three negative cultures in the last 8 months of treatment and without any evidence of failure. Treatment complete: treatment completed without the documentation of three consecutive negative cultures after the intensive phase, with no evidence of failure and no signs of active disease. Failure: the need for treatment change for at least two classes of anti-TB drugs or treatment termination due to any of the following reasons: lack of monitoring culture conversion, resistance amplification, bacteriological reversion, or clinical decision, owing to poor treatment response or adverse drug reactions. Lost to follow-up (LFU): patients with interrupted treatment for two or more consecutive months. Not evaluated: patients with unknown or undocumented treatment outcomes. Died: a patient who died during treatment. Favorable treatment outcomes: the study participants with the treatment outcome as cured and completed were grouped under the term favorable treatment outcomes (Asemahagn, 2021). Unfavorable treatment outcomes: treatment outcomes such as death, lost to follow-up, transferred out, and failure were categorized under unfavorable treatment outcomes.

Sputum smear converted: the group of patients whose sputum smear has been converted to negative following a specific duration of anti-TB treatment. It is validated by two consecutively taken negative smears 30 days apart (Asemahagn, 2021). Sputum culture converted: the group of patients whose sputum culture has been converted to negative, confirmed by two continuous negative cultures spaced over a period of 30 days. In this study, sputum smear and culture were tested every month. Initial sputum smear and culture conversion are the negative results at the completion of the first month of treatment. The study participants were also stratified into two groups based on the sputum smear conversion in the first month: converted and not converted. Similar was the case with culture conversion. Baseline tests: any tests performed before the start of the treatment among study participants.

### Statistical analysis

Before entering the data, it was cleaned and validated for consistency. The data were input and analyzed using SPSS version 26. For categorical variables, proportions were calculated and expressed as percentages, and for continuous variables, means and medians were stated. The Kaplan–Meier (KM) method was applied to determine the time for sputum smear and culture conversion separately, and the patients were stratified into favorable and unfavorable treatment outcomes. The censored event included patients without a sputum smear or culture conversion. The survival time difference between different strata was assessed using the log-rank test. The associations of independent variables with first-month sputum and first-month culture conversions as well as factors associated with treatment outcomes were evaluated based on binary and multiple logistic regression (LR) analysis. For multiple regressions, the predictors with a *p*-value ≤0.05 and a crude/unadjusted odds ratio (uOR) were selected for further analysis. The 95% confidence intervals (CI) and adjusted odds ratio (aOR) were computed. The analysis of the receiver operating characteristics curve (ROC) was done to see the extent to which the anticipated model could discriminate amongst real outcomes. The Hosmer–Lameshow (H-L) test was performed to determine the calibration of the resultant multivariable LR model. Variables were deemed statistically significant when their double-sided *p*-value was less than or equal to 0.05.

## Results

### Sociodemographic characteristics of study participants

A total of 902 bacteriologically confirmed DR-TB cases started getting treatment for the disease from October 2014 to August 2023. One hundred and sixty-six patients were excluded from the study on account of incomplete medical records, extrapulmonary TB, pregnancy, and HIV status. The data of 736 patients who matched the inclusion criteria were employed in the conclusive analysis.

A slightly higher percentage (53.4%) of study participants comprises males (N = 393) with a mean weight of 47.76 ± 11.97 kg. Among different age groups, the participants aged <25 or >44 years were of equal proportion, with a mean age of 36.5 ± 16.5 years. Over 50 % of the study subjects were unemployed (52.9%), while among the employed ones, the majority were laborers, with a percentage of 27.6% overall. A higher percentage of study participants (63%) were living in rural residences in the city. In addition, almost one-third of the study participants were smokers and alcoholics, while some were observed to use Naswar at baseline, as illustrated in Table 1.

**TABLE 1:** Sociodemographic characteristics of the study participants.

Characteristic	Total N (%)
Gender	
Female	343 (46.6%)
Male	393 (53.4%)
Age* (36.5 ± 16.5 years)	
<25	238 (32.3%)
25–34	138 (18.8%)
35–44	125 (17%)
>44	235 (31.9%)
Weight*	47.76 ± 11.97
Residential area	
Rural	464 (63%)
Urban	272 (37%)
Occupation	
Unemployed	389 (52.9%)
Student	118 (16%)
Labor	203 (27.6%)
Businessman	26 (3.5%)
Behavioral status	
Tobacco use	252 (34.2%)
Alcohol use	16 (2.2%)
Naswar	20 (2.7%)

Categorical data are displayed as percentages while the continuous factors* are represented as the mean ± SD.

### Clinico-pathological characteristics of the study participants

The family history of TB disease was only observed in 57 (7.7%) of the study subjects. Almost one-third of the included patients were suffering from comorbidities, with a greater percentage of hypertension (19.6%). A total of 55.3% of the cases were observed with a previous history of TB disease, with a greater proportion (77.8%) present with evidence of treatment category I. Among the previously treated patients, a total of 39.9% of the patients were present with treatment not evaluated, while the previous treatment of 8.6% and 2% of the study participants was observed as failed and cured, respectively. A total of 63 patients (8%) were observed to have been previously treated with SLD. The duration of sickness of research participants ranged from less than 6 months (36%) to more than 2 years (5.4%) prior to the disease diagnosis.

Of the 736 included cases, 526 (71.7%) were diagnosed with MDR-TB, while rifampicin resistance was observed in 21.6% of the research subjects. A small proportion of study participants were diagnosed with XDR (1.9%), and two cases were also present with poly-resistant TB.

Among the bacteriological testing of the study participants, 1+ sputum smear and 1+ culture were the most frequent, with a proportion of 477 (64.8%) and 440 (59.8%), respectively. A total of 29 patients (3.9%) had shown negative smears but positive cultures, while negative cultures with positive smear test results were also found in 120 (16.3%) of the participants.

Upon the radiological examination, the highest percentage of study participants was observed with cavitary disease (84.2%), and the chest X-ray showed bilateral disease among 64% of the patients, with 2–3 zones involved in the majority of the cases (544, 74%), as illustrated in Table 2.

**TABLE 2:** Clinico-pathological characteristics of the study participants.

Characteristic	N (%)
Family history of TB	57 (7.7%)
Comorbidities	
Diabetes	144 (19.6%)
Hypertension	45 (6.1%)
Others	42 (5.7%)
Previous history of TB disease	407 (55.3%)
Previous treatment category	
Cat I	317 (43.1%)
Cat II	90 (12.2%)
Previous treatment outcomes	
Cured	15 (2%)
Complete	13 (1.8%)
LFU	14 (1.9%)
Relapse	8 (1.1%)
Treatment not evaluated	294 (39.9%)
Failed	63 (8.6%)
History of SLD use	59 (8%)
Duration of sickness prior to diagnosis	
<6 months	265 (36%)
6–12 months	376 (51.1%)
13–24 months	55 (7.5%)
>24 months	40 (5.4%)
Type of resistance	
MDR	528 (71.7%)
XDR	14 (1.9%)
RR	192 (26.1%)
Poly-drug-resistant	2 (0.3%)
Baseline sputum smear microscopy	
1 positive	477 (64.8%)
2 positive	193 (26.2%)
3 positive	37 (5%)
Smear negative but culture positive	29 (3.9%)
Baseline culture test results	
1 positive	440 (59.8%)
2 positive	138 (18.47%)
3 positive	38 (5.2%)
Culture negative but smear positive	120 (16.3%)
Chest X-ray	
Unilateral	265 (36%)
Bilateral	471 (64%)
Extent of disease	
1 zone	118 (16%)
2–3 zones	544 (74%)
4–6 zones	74 (10%)
Cavitation	620 (84.2%)
Lung lesions on chest X-ray	621 (84.4%)

Categorical data are displayed as percentages. Abbreviations and acronym: Cat I, category I; Cat II, category II; LFU, lost to follow-up; SLD, second-line drugs; MDR, multidrug resistant; XDR, extensively drug resistance; RR, rifampicin resistance.

### Pattern of resistance to anti-TB drugs among the study participants

The pattern of resistance to drugs used in TB treatment was also assessed through DST, as shown in Table 3. Among the anti-TB drugs, isoniazid was the most resistant drug (73.6%) among study participants, with cefazolin (0.7%) and bedaquiline (0.8%) being the least resistant drugs. Fluoroquinolones have shown an almost equal proportion of resistance (ofloxacin = 34.4% and levofloxacin = 35.1%), except moxifloxacin with a little less percentage (24.3%).

**TABLE 3:** Pattern of resistance to anti-TB drugs among the study participants.

Drug	N (%)
Isoniazid	542 (73.6%)
Pyrazinamide	152 (20.7%)
Ethambutol	245 (33.3%)
Streptomycin	168 (22.8)
Kanamycin	16 (2.2%)
Amikacin	21 (2.9%)
Ofloxacin	253 (34.4%)
Levofloxacin	258 (35.1%)
Moxifloxacin	179 (24.3%)
Bedaquiline	6 (0.8%)
Cefazolin	5 (0.7%)
Linezolid	8 (1.09%)

Categorical data are displayed as percentages.

### Treatment characteristics of the study participants

Of the included patients, 44.7% of the cases were registered as new treatment groups, while a total of 294 (39.9%) cases were grouped under others previously treated. The year 2021 saw the majority of enrolled patients (19%), with almost an equal proportion enrolled in 2015 (10.9%), 2016 (10.5%), and 2020 (11.4%). A total of 581 (78.9%) of the study participants were treated with long-term regimens. Upon the completion of the treatment, a total of 63.2% of patients were observed to have favorable treatment outcomes with a cure rate of 61.1%. Overall, among the patients with unfavorable treatment outcomes, 16.6% died and 12.2% were lost to follow-up. The mean treatment duration was 15 ± 7.88 months, as illustrated in Table 4.

**TABLE 4:** Treatment characteristics of the study participants.

Characteristic	N (%)
Registration group	
New	329 (44.7%)
Previously treated after relapse	36 (4.9%)
Previously treated after failure	63 (8.6%)
Previously treated after LFU	14 (1.9%)
Others previously treated	294 (39.9%)
Year of treatment started	
2014	8 (1.1%)
2015	80 (10.9%)
2016	77 (10.5%)
2017	102 (13.9%)
2018	95 (12.9%)
2019	105 (14.3%)
2020	84 (11.4%)
2021	140 (19%)
2022	45 (6.1%)
Regimen of treatment	
STR	155 (21.1%)
LTR	581 (78.9%)
Duration/length of treatment (months)	15 ± 7.88
First-month sputum smear conversion	435 (59.1%)
First-month sputum culture conversion	395 (53.7%)
Sixth-month sputum smear conversion	582 (79.1%)
Sixth-month sputum culture conversion	581 (78.9%)
Endpoint sputum smear conversion	581 (78.9%)
Endpoint sputum culture conversion	572 (77.7%)
Mean time to sputum conversion (months)	1.29 ± 1.457
Mean time to culture conversion (months)	1.29 ± 1.423
Outcomes of treatment	
Cured	451 (61.3%)
Completed	14 (1.9%)
Failure	35 (4.8%)
Transfer out	25 (3.4%)
LFU	89 (12.1%)
Died	122 (16.6%)
Treatment success rate	
Favorable	465 (63.2%)
Unfavorable	271 (36.8%)

Categorical data are displayed as percentages. Abbreviations and acronym: STR, short-term regimen; LTR, long-term regimen.

### Time to sputum smear conversion among the study participants

**FIGURE 2:**
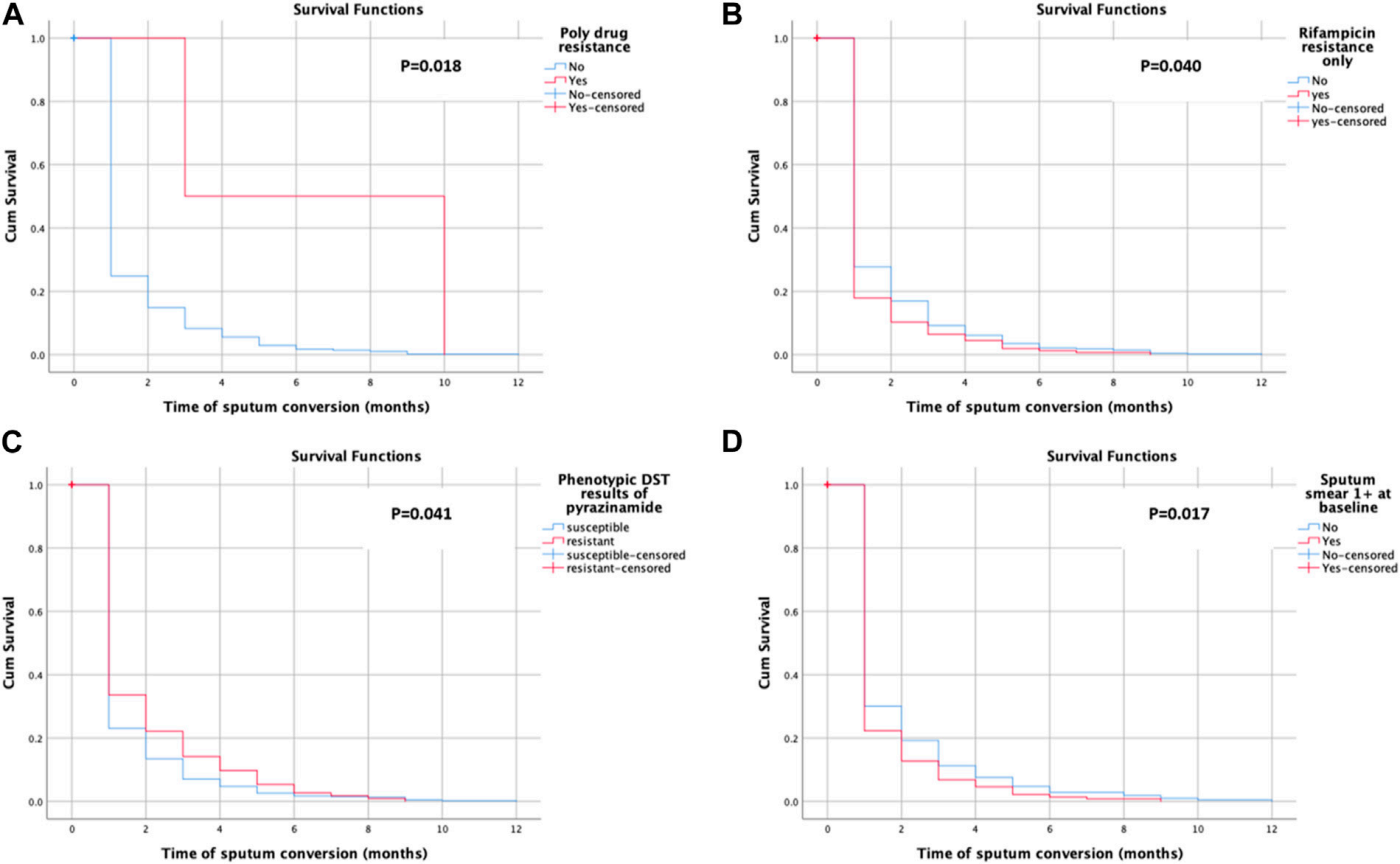
Kaplan–Meier plot of time to sputum smear conversion.

Sputum smear conversion was observed among 581 (78.9%) of the participants. The patients underwent a follow-up for a total of 11,037 person-months (919.75 person-years) (note: the person time is the sum of the total duration of the treatment of study participants). The mean time for sputum smear conversion was 1.29 ± 1.46 months. The mean time for sputum smear conversion in poly-drug-resistant cases (6.50 ± 4.95 months), rifampicin-resistant only cases (1.17 ± 1.22 months), pyrazinamide-resistant cases (1.41 ± 1.65 months), and patients with 1 +ve sputum smear case (1.26 ± 1.81 months) was also calculated. The KM survival curve revealed the quicker and steepest time to culture conversion within an initial month and a half, with a little change subsequently, as shown in Figure 2. Figure 2B shows the average likelihood of survival on time to sputum smear conversion among patients with rifampicin resistance only with the following values: 0.179 after the first, 0.103 after the second, 0.064 after the third, 0.045 after the fourth, 0.019 after the fifth, 0.013 after the sixth, and 0.006 after the seventh month. The average likelihood of survival on time to sputum smear conversion among the pyrazinamide-resistant group was 0.336 after the first, 0.221 after the second, 0.142 after the third, 0.097 after the fourth, 0.053 after the fifth, 0.027 after the sixth, 0.018 after the seventh, and 0.009 after the eighth month, as shown in Figure 2C.

### Time to culture conversion among the study participants

**FIGURE 3:**
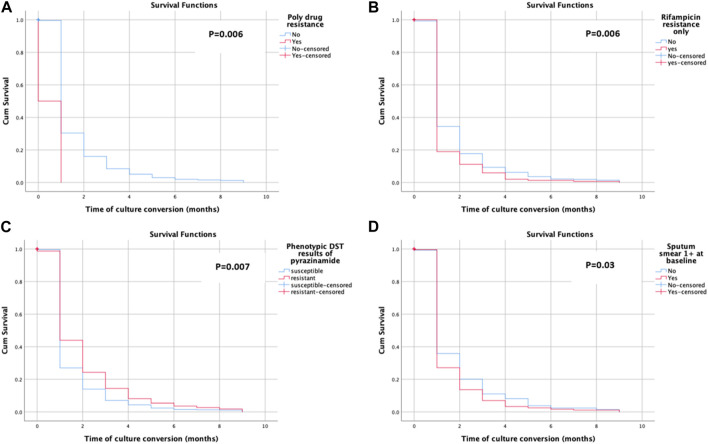
Kaplan–Meier plot of time to sputum culture conversion.

Sputum culture conversion was observed among 572 (77.7%) of the study subjects. The mean time for the sputum culture conversion was 1.29 ± 1.42 months. The mean time for the sputum smear conversion in poly-drug-resistant cases (0.50 ± 0.71 months), rifampicin-resistant only cases (1.13 ± 1.14 months), pyrazinamide-resistant cases (1.49 ± 1.72 months), and patients with 1 positive sputum smear case (1.19 ± 1.31 months) was also calculated. The KM survival curve showed the quicker and steepest time to culture conversion within the initial month and a half, with a resultant little change, as shown in Figure 3. The average likelihood of survival on time to sputum culture conversion among the rifampicin-resistant only group was 0.190 after the first, 0.111 after the second, 0.059 after the third, 0.020 after the fourth, 0.013 after the fifth, and 0.007 after the seventh month, as shown in Figure 3A. Figure 3D shows the average likelihood of survival on time to sputum culture conversion among patients with 1 positive sputum smear case with the following values: 0.271 after the first, 0.136 after the second, 0.069 after the third, 0.033 after the fourth, 0.025 after the fifth, 0.017 after the sixth, and 0.011 after the seventh month.

### Determinants of initial sputum smear conversion among the study participants

Overall, 435 (59.1%) converted their sputum smear in the initial month of treatment. Univariate analysis indicated that the males with a mean weight of 47.7 ± 11.97 kg, students with a present or previous history of smoking and tobacco use, with the previous treatment outcome as failure or not evaluated, registered under treatment after the failure group, with cavitation and lung lesions on chest X-ray, having sickness of more than a year, belonging to the RR only group, with three positive or negative culture results at baseline, with first-month culture conversion, and negative sputum smear at culture results at the sixth month, favorable treatment outcomes, duration of treatment and cured, died and LFU, as treatment outcomes were the predictors of initial sputum smear conversion.

**FIGURE 4:**
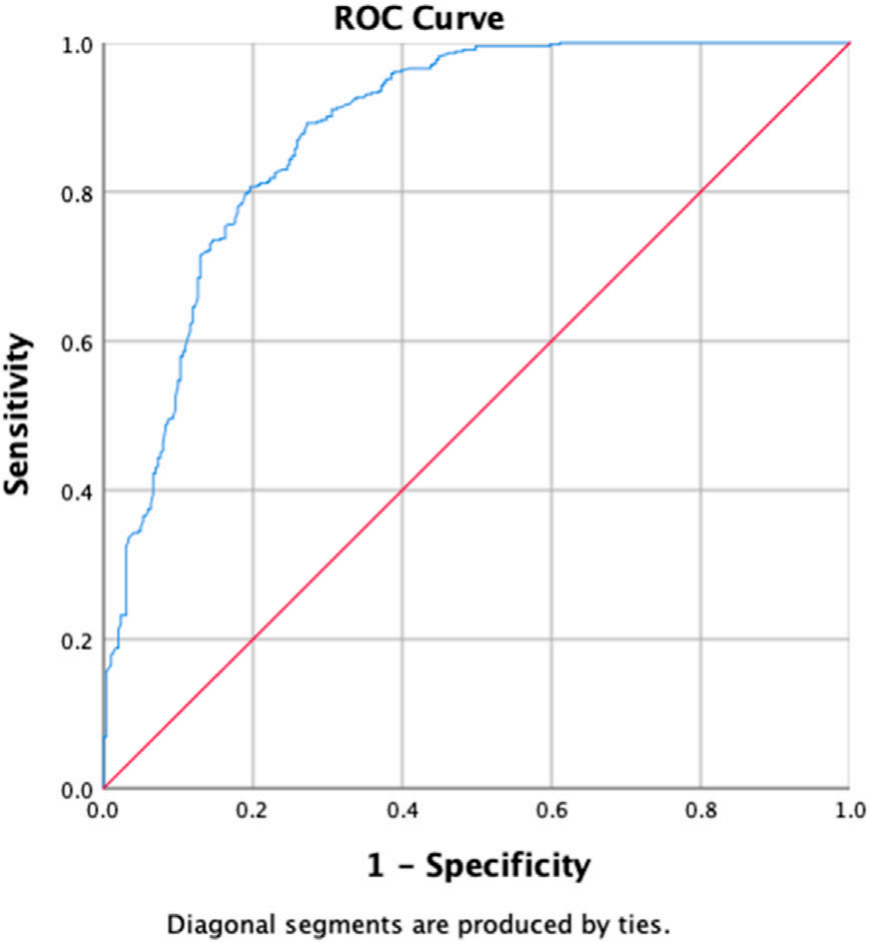
Receiver operating characteristics curve analysis of the multivariate logistic model predicting the determinants of first-month sputum conversion (area under the curve, AUC: 0.881, (95% CI [0.855–0.907]; *p* < 0.001)).

The uOR for these predictors is mentioned in Table 5. In the multivariate analysis, students (*p*-value = 0.029, aOR = 0.496, 95% CI = 0.265–0.930), the group with previously not evaluated treatment (*p*-value = 0.039, aOR = 0.618, 95% CI = 0.391–0.977), first-month culture conversion (*p*-value = <0.001, aOR = 5.739, 95% CI = 3.700–8.900) and final culture conversion the completion of treatment (*p*-value = <0.001, aOR = 0.135, 95% CI = 0.046–0.395) were strongly correlated with initial sputum smear conversion as shown in Table 5. We observed that the strongest determinant of the initial sputum smear conversion was the first-month culture conversion (aOR = 5.739). Being students, previously unevaluated treatment and endpoint culture conversion are negatively associated with the initial sputum smear conversion. The H–L test statistics have depicted χ2 = 12.84, degree of freedom = 8, and *p*-value = 0.118, indicating the identical predicted and observed (model) values of the response variable and model are excellent fits to the data. ROC curve analysis with an AUC of 0.881 (95% CI = 0.855–0.907; *p* < 0.001) has shown the outstanding predictive ability of the LR model for the initial sputum smear conversion, as shown in Figure 4.

**TABLE 5:** Univariate and multivariate logistic regression analysis of determinants of initial sputum smear conversion among the study participants.

Variable	Univariate			Multivariate		
	*p-*value	uOR	95% CI	*p-*value	aOR	95% CI
Weight (≤50 kg)	.019	1.469	1.067–2.022			
Gender (female)	.046	0.740	0.550–0.995			
Student	0.013	0.585	0.383–0.893	0.029	0.496	0.265–0.930
Smoking	0.002	1.639	1.204–2.231			
Naswar	0.033	2.760	1.088–7.001			
Previously treated after failure	0.03	2.205	1.304–3.727			
Previous treatment not evaluated	0.020	0.697	0.515–0.945	0.039	0.618	0.391–0.977
Registration group as failure	0.003	2.205	1.304–3.727			
Chest X-ray cavitatory	0.019	1.659	1.085–2.536			
Lung lesions on chest X-ray	0.014	1.713	1.117–2.627			
Duration of sickness (≤ 1 year)	<0.001	2.321	1.497–3.599			
Rifampicin-resistant only	0.013	0.648	0.459–0.914			
Baseline culture 3+ve	0.021	2.167	1.124–4.174			
Culture negative	0.002	0.517	0.337–0.793			
First-month culture conversion	<0.001	14.916	10.342–21.513	<0.001	5.739	3.700–8.900
Endpoint culture conversion	<0.001	0.017	0.008–0.036	<0.001	0.135	0.046–0.395
Sputum smear conversion at the sixth month	<0.001	0.035	0.019–0.063			
Sputum culture conversion at the sixth month	<0.001	0.038	0.021–0.067			
Treatment outcome favorable	<0.001	0.130	0.093–0.182			
Duration of treatment (<1 year)	0.001	0.588	0.436–0.793			
Cured	<0.001	0.134	0.096–0.187			
LFU	<0.001	2.359	1.507–3.722			
Died	<0.001	5.114	3.318–7.883			

Hosmer–Lemeshow (H–L) test statistics: χ2 = 12.84, degree of freedom = 8, and *p-*value = 0.118. Only significant association is displayed. LFU, lost to follow-up.

### Determinants of initial culture conversion among the study participants

**FIGURE 5:**
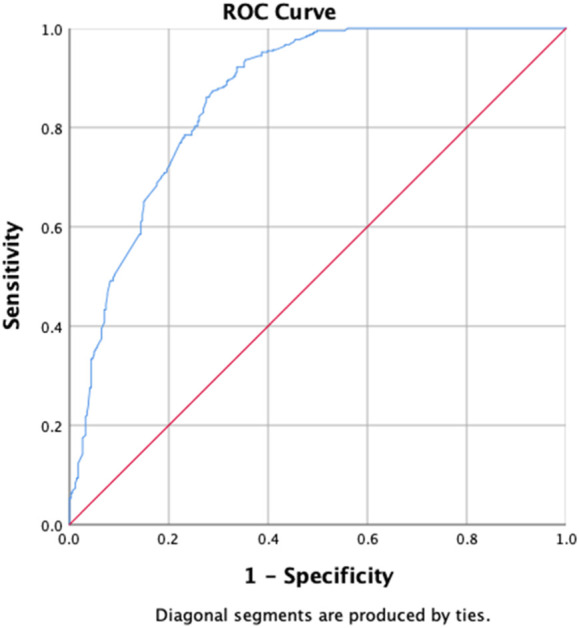
Receiver operating characteristics curve analysis of the multivariate logistic model predicting the determinants of initial culture conversion (area under the curve, AUC: 0.862, (95% CI [0.835–0.889]; *p* = 0.014)).

Table 6 shows the results of the determinants of the initial culture conversion of the study participants, with the uOR ranging from 0.031 to 14.916. Diabetes (*p-*value = 0.002, aOR = 0.448, and 95% CI = 0.266–0.752), zone 1 extent of disease upon radiology (*p*-value = 0.033, aOR = 0.521, and 95% CI = 0.287–0.947), first-month sputum conversion (*p-*value < 0.001, aOR = 7.446, and 95% CI = 4.869–11.388), and LFU as treatment outcomes (*p-*value = 0.008, aOR = 0.248, and 95% CI = 0.089–0.691) are the determinants of the initial culture conversion. The first-month conversion of the sputum smear is seven times more strongly associated with the initial culture conversion. The H–L test statistics have depicted χ2 = 17.597, degree of freedom = 8, and *p-*value = 0.024, indicating that the model is adequately fit. ROC curve analysis with an AUC of 0.862 (95% CI [0.835–0.889]; *p* = 0.014) has shown outstanding predictive ability for the LR model for the initial sputum culture conversion, as shown in Figure 5.

**TABLE 6:** Univariate and multivariate logistic regression analysis of determinants of time to sputum culture conversion among the study participants.

Variable	Univariate			Multivariate		
	*p-*value	uOR	95% CI	*p-*value	aOR	95% CI
Gender (female)	0.012	0.689	0.514–0.922			
Previous SLD use	0.039	1.768	1.029–3.037			
Diabetes	0.006	0.593	0.407–0.863	0.002	0.448	0.266–0.752
Smoking	0.001	1.676	1.233–2.277			
Extent of disease 1 zone	0.05	0.671	0.448–1.004	0.033	0.521	0.287–0.947
Zone 4–6	0.034	0.590	0.363–0.960			
Chest X-ray cavitation	0.030	1.571	1.044–2.364			
Lung lesions on chest X-ray	0.022	1.614	1.070–2.434			
Duration of sickness (≤1 year)	0.002	1.983	1.276–3.082			
MDR	0.002	1.701	1.224–2.364			
RR	<0.001	0.544	0.387–0.765			
Baseline culture negative	0.002	0.523	0.347–0.789			
First-month sputum converted	<0.001	14.916	10.342–21.513	<0.001	7.446	4.869–11.388
Sputum smear conversion at the sixth month	<0.001	0.032	0.016–0.063			
Sputum culture conversion at the sixth month	<0.001	0.031	0.016–0.062			
Treatment outcome favorable	<0.001	0.173	0.125–0.241			
Cured	<0.001	0.168	0.121–0.234			
LFU	0.028	1.652	1.056–2.585	0.008	0.248	0.089–0.691
Died	<0.001	5.263	3.333–8.312			

Hosmer–Lemeshow (H–L) test statistics: χ2 = 17.597, degree of freedom = 8, and *p*-value = 0.024. Only significant association is displayed. Abbreviation and acronym: SLDs, second-line drugs; MDR, multidrug resistance; RR, rifampicin resistance; LFU, lost to follow-up.

### Factors associated with treatment outcomes among the study participants

**FIGURE 6:**
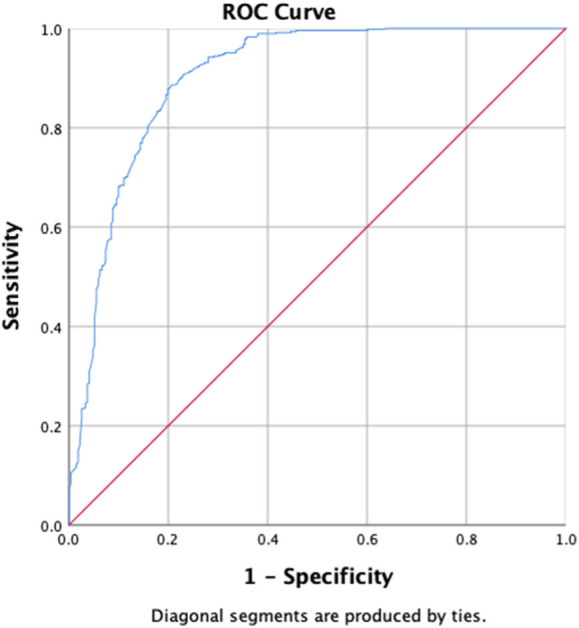
Receiver operating characteristics curve analysis of the multivariate logistic model predicting the factors associated with treatment outcomes (area under the curve, AUC: 0.902, (95% CI [0.877–0.927]; *p* < 0.001)).

Upon univariate analysis, age, gender, student, labor, unemployed, previous treatment, with SLD, smoking, alcohol and Naswar use, lung lesions and cavitation on chest X-ray, duration of sickness ≤1 year, XDR, 3 positive baseline smearcases, negative baseline culture, first-month sputum conversion, first-month culture conversion, endpoint culture conversion, sputum smear conversion at the sixth month, sputum culture conversion at the sixth month, duration of treatment (<1 year) have shown the uOR ranging from 0.006 to 7.705, as shown in Table 7. In multivariate analysis, previous treatment with SLD (*p-*value = 0.046, aOR = 2.436, and 95% CI = 0.956–6.208), smoking (*p-*value = 0.001, aOR = 2.556, and 95% CI = 1.439–4.434), duration of sickness (*p-*value = 0.04, aOR = 2.113, and 95% CI = 1.033–4.324), XDR (*p-*value = 0.034, aOR = 6.985, and 95% CI = 1.159–42.109), first-month sputum conversion (*p-*value = 0.029, aOR = 1.748, and 95% CI = 1.060–2.884), endpoint culture conversion (*p-*value < 0.001, aOR = 0.010, and 95% CI = 0.003–0.036), and duration of treatment (*p-*value < 0.001, aOR = 0.340, and 95% CI = 0.214–0.542) are the factors correlated with treatment outcomes among the research participants. Factors such as the previous use of SLD, smoking, duration of sickness, and first-month sputum conversion are almost equally associated with favorable treatment outcomes. However, XDR-TB is six times strongly more associated with favorable treatment outcomes in our study. The longer durations of treatment and endpoint culture conversion are negatively associated with favorable treatment outcomes. The H–L test statistics have displayed χ2 = 5.964, degree of freedom = 8, and *p-*value = 0.651, indicating that the model is strongly related to the data. ROC curve analysis with an AUC of 0.902 (95% CI [0.877–0.927]; *p* < 0.001) has shown outstanding predictive ability for the LR model for the initial sputum culture conversion, as shown in Figure 6.

**TABLE 7:** Univariate and multivariate analysis of factors associated with treatment outcomes among the study participants.

Variable	Univariate			Multivariate		
	*p-*value	uOR	95% CI	*p-*value	aOR	95% CI
Age (≤37 years)	0.000	1.929	1.424–2.613			
Gender (female)	0.029	0.714	0.527–0.966			
Unemployed	<0.001	1.891	1.392–2.568			
Labor	0.019	0.661	0.467–0.935			
Student	0.010	0.561	0.361–0.872			
Previous treatment complete	0.05	0.140	0.018–1.081			
Previous SLD use	<0.001	3.740	2.132–6.561	0.05	2.436	0.956–6.208
Smoking	<0.001	2.399	1.751–3.286	0.001	2.526	1.439–4.434
Alcohol	0.039	2.931	1.053–8.157			
Naswar	0.001	5.391	1.937–15.00			
Chest X-ray cavitation	0.001	2.262	1.420–3.601			
Lung lesions on chest X-ray	<0.001	2.362	1.474–3.784			
Duration of sickness (≤1 year)	<0.001	2.705	1.744–4.196	0.04	2.113	1.033–4.324
XDR	0.013	4.416	1.371–14.22	0.034	6.985	1.159–42.11
Baseline smear 3 positive	0.041	1.979	1.028–3.810			
Baseline culture negative	0.007	0.546	0.352–0.847			
First-month sputum converted	<0.001	7.705	5.498–10.79	0.029	1.748	1.060–2.884
First-month culture converted	<0.001	5.767	4.144–8.027			
Endpoint culture conversion	<0.001	0.006	0.002–0.017	<0.001	0.010	0.003–0.036
Sputum smear conversion at the sixth month	<0.001	0.046	0.028–0.076			
Sputum culture conversion at the sixth month	<0.001	0.045	0.027–0.075			
Duration of treatment (<1 year)	<0.001	0.351	0.258–0.479	<0.001	0.340	0.214–0.542

Hosmer–Lemeshow (L–R) test statistics: χ2 = 5.964, degree of freedom = 8, and *p-*value = 0.651. Only significant association is displayed. Abbreviations and acronym: SLD, second-line drug; XDR, extensively drug resistant.

## Discussion

According to the information we have, this is the first-ever investigation assessing the determinants of initial sputum smear, culture conversion, and subsequent treatment outcomes among DR-TB cases in Pakistan. Given the rapid development of resistance against anti-TB drugs, the efficacy and success of treatment remain areas of concern. Such concerns are even more profound for patients with longer treatment durations. Following the start of treatment, sputum smears and culture negativity are essential markers in assessing the success of anti-TB treatment in underdeveloped nations.

Most of the patients in our study were male (53.4%), similar to previous studies (Germossa, 2017; Tola et al., 2019; Muñoz-Sellart et al., 2010). Males may have a greater incidence of TB than females because they are more likely than females to be exposed to the illness or to use healthcare facilities. Due to financial limitations, female may also understate their illnesses and seek help elsewhere than in medical facilities. The mean age of study participants was almost 37 years, which is consistent with the previous study (Saifullah et al., 2021).

Our analysis revealed that the median time to sputum smear conversion was 1 month, which is comparable to previous studies with a median time of conversion of 35 (Parikh et al., 2012) and 34 days (Tekalegn et al., 2020). The current findings were also found to be greater than previous studies with a median time of 24 (Bisognin et al., 2019) and 21 days (Asemahagn, 2021) and lower than a study with a median time of 60 days (Tekalegn et al., 2020) of sputum conversion. Such discrepancies could be attributed to variances in socio-economic standings, selected study period, duration of follow-up, clinical characteristics of the study subjects, and strength of the TB control program. All these differences can influence the time to sputum smear conversion in PTB cases.

The median time to initial culture conversion was 1 month, less than the findings of other studies with a median time of 56 days (Kim et al., 2016), 59 days (Tierney et al., 2014; Rieu et al., 2016), 60 days (Rodriguez et al., 2013; Putri et al., 2014), 61 days (Tekalegn et al., 2020), 62 days (Assemie et al., 2020), 91 days (Rieu et al., 2016; Velayutham et al., 2016), and 93 days (Shah et al., 2008). However, our findings were greater than a study showing a median time of sputum smear conversion of 15 days (Kim et al., 2016). In contrast, other studies might have used different time periods to check for sputum smears or culture conversion (Weldemhret et al., 2023; Kurbatova et al., 2012; Liu et al., 2018; Horne et al., 2010). By reducing the need for injectable drugs, rapid sputum culture conversion cannot only simplify the treatment regimen but also reduce vestibular and auditory toxicity and improve patient comfort. In addition, it can also stop the transmission of disease from infected individuals to family members, the community, and healthcare providers.

Delayed conversion of the sputum smear or culture might result in poor treatment outcomes. Therefore, the timely identification of determinants of sputum smear and culture conversion can improve the overall treatment response and success of the infection control program in the country.

Almost 80% of the patients have shown sputum smear and culture conversion just over 6 months after treatment initiation. These findings are closer to the findings of previous studies: 81.4% (Tierney et al., 2014) for culture, 89% for culture, and 94% for sputum smear (Alene et al., 2018). The K–M test has shown the quickest and steepest time to sputum smears and culture conversion within 2 months following the start of anti-TB treatment. The survival distribution among the patients with poly-drug-resistant (*p* = 0.018), rifampicin-resistant only groups (*p* = 0.040), pyrazinamide-resistant (*p* = 0.041), and 1 positive initial sputum smear *bacillus* load (*p* = 0.017) was of statistical significance. The survival curves have shown that there is a slight variation in survival time to initial sputum smear and culture conversion after half a year, supporting the treatment guidelines of the WHO (Weldemhret et al., 2023; Organization, 2013; Organization, 2014). The possibility that the patient’s immune system is weakened and that they are unable to undergo sputum/culture conversion with any medication plan is one explanation for the reduced conversion rate later, even if this requires more thorough prospective research. The reduced conversion rate after the stated months might result in the transmission of disease to communities with poor infection control prevention programs, in particular. The median time for sputum smear conversion in poly-drug-resistant cases (90 days) increased as compared to non-poly-drug-resistant cases. A possible explanation for the delayed sputum smear conversion among poly-drug-resistant cases might be previous unsuccessful treatment, high bacterial load, and poor response to the SLD.

Sputum smear conversion at the first month was evident in 59.1% of patients, while a total of 54.7% patients showed first-month culture conversion upon microbiological testing. In our study, the first-month culture conversion was strongly associated with the first-month sputum smear conversion among the participants. Although the determinant of the first-month sputum culture conversion was the first-month sputum smear conversion, our findings are contrary to the previous study showing new treatment cases, HIV-positive patients, and patients with 1 positive sputum smear as factors related to initial sputum culture conversion (Weldemhret et al., 2023). However, another study showed that older age, a high bacterial load at baseline, and the severity of lung involvement are responsible for delayed sputum smear conversion (Kurbatova et al., 2012).

In the present investigation, the treatment success rate was 63.2%, in contrast to previous studies with a treatment success rate of 95% (Endris et al., 2014), 92.5% (Tola et al., 2019), 89% (Berhe et al., 2012), 88% (Tadesse and Tadesse, 2014), and 87.1% (Melese et al., 2016). The moderate treatment success rate in this investigation might be related to the inclusion of a larger number of cases with previous disease and treatment histories as well as resistance toward anti-TB treatment.

Previous treatment with SLD, previous history of smoking, duration of sickness less than a year, XDR-TB, and first-month sputum conversion are the factors responsible for the favorable treatment outcomes among the study participants. Our observations are similar to previous publications showing negative smears after the second month (Chaves Torres et al., 2019). Negative smears after the end of the second month, <65 years of age, nonalcoholic, HIV negative, new treatment cases (Tola et al., 2019), non-smokers, urban residents, females, non-diabetics, PTB patients, and positive smears on admission are the determinants of successful treatment outcomes (Chaves Torres et al., 2019). In an investigation conducted in Ethiopia, female patients with a baseline weight of 20–29 kg were HIV-negative, and new treatment cases were the factors responsible for favorable treatment outcomes (Tola et al., 2019).

### Study limitations and strengths

The findings of this study should be viewed in the context of a few shortcomings. First, its retrospective nature and being a monocentric study might have resulted in some missing data and subjective evaluation of certain variables. Second, there were no patients included with a positive HIV status, which makes it impossible to extrapolate the results for PLHIV (people living with HIV). Last but not least, our analysis is backed by the first-ever study analyzing the sputum smear and culture conversion, time to sputum smear and culture conversion, and determinants of conversion and predictors of favorable outcomes in a single study in Pakistan.

## Conclusion

Our findings indicated that the mean sputum smear and culture conversion happened in the initial two months. The first-month culture conversion was the factor associated with the initial smear conversion, whereas the initial culture conversion was associated with the first-month sputum smear conversion. Previous treatment with SLD, smoking history, shorter duration of illness, XDR-TB, and first-month sputum smear conversion were the predictors of favorable treatment outcomes. Growing resistance patterns in both treatment-naïve and previously treated groups raised concerns about NTP’s inefficiency and ineffectiveness. The identification of the variables linked to sputum smear and culture conversion and treatment outcomes will yield significant benefits in terms of appropriate treatment and preventive and management decisions. The consideration of these determinants, rapid diagnosis, a suitable therapeutic regimen, coordinated implementation of DOTS, and clarified infection and transmission control methods ought to be in place.

## Data Availability

The original contributions presented in the study are included in the article/supplementary material; further inquiries can be directed to the corresponding authors.
